# Access to Care in Lyme Disease: Clinician Barriers to Providing Care

**DOI:** 10.3390/healthcare10101882

**Published:** 2022-09-27

**Authors:** Lorraine B. Johnson, Elizabeth L. Maloney

**Affiliations:** 1 Principal Investigator, MyLymeData, LymeDisease.org, San Ramon, CA 94583, USA; 2 Partnership for Tick-Borne Diseases Education, P.O. Box 84, Wyoming, MN 55092, USA

**Keywords:** persistent Lyme disease, chronic Lyme disease, access to care, health equity, health disparities, physician survey, structural barriers to care

## Abstract

Patients with persistent Lyme disease/chronic Lyme disease (PLD/CLD) encounter significant barriers to accessing medical care. Although this health inequity has been explored from the patient perspective, the obstacles clinicians encounter when providing care to this group of patients have not been examined. The primary goal of this study was to identify the challenges faced by clinicians who provide care for patients with PLD/CLD. Clinicians who treat PLD/CLD were surveyed regarding their professional backgrounds, general challenges to providing care, supply and demand constraints, insurance restrictions, and regulatory and legal challenges. Clinicians treating patients with PLD/CLD have developed substantial clinical expertise but encounter multiple clinical, regulatory and financial impediments to providing care. Clinician-encountered barriers may be powerful disincentives for providing care patients with PLD/CLD and make it difficult to retain and recruit clinicians who will care for the rapidly expanding PLD/CLD populations. Understanding these barriers and identifying potential solutions is essential to resolving the current supply/demand imbalance that makes it difficult for patients to receive the care they need to become well.

## 1. Introduction

Lyme disease is an increasingly important public health threat, with an estimated 476,000 new cases per year in the U.S. [1]. The infection, caused by *Borrelia burgdorferi* and acquired via the bite of infected *Ixodes* spp. ticks, can be multi-staged and frequently produces a multi-systemic illness. In early (acute) Lyme disease, the infection is localized to the skin. Late disease results from bacterial dissemination to other body sites, most commonly to joints and the nervous system [2]. If diagnosed and treated early in the course of the infection, the majority of Lyme disease cases resolve without complications but treatment failures do occur with early treatment and many patients are not diagnosed until they have late disease [3,4,5]. 

Complete recovery is more likely for patients with early disease and delayed treatment is associated with an increased risk of remaining ill after an initial course of antibiotics [6,7,8]. Reported failure rates in patients with late neurologic disease have been quite high [9,10]; for example, 61% of subjects treated with 30 days of IV ceftriaxone for Lyme encephalopathy failed to return to their pre-Lyme health status [10]. While patients with early disease fare better, even in an ideally treated cohort of 234 patients who received appropriate oral antibiotics, 44% remained symptomatic alone (at moderate to severe levels) or remained symptomatic with functional impairments [11]. 

In this paper, we refer to patients who have been diagnosed with Lyme disease by a clinician and who remain ill six or more months following antibiotic treatment as having persistent or chronic Lyme disease (PLD/CLD). As such, cases of PLD/CLD can result from patients who were treated for either early or late disease yet remain ill [4,6,12,13,14,15,16] Although the prevalence of PLC/CLD is unknown, a 2019 paper predicted slightly less than 2 million cases in 2020 [17].

Significant uncertainty regarding the diagnosis and treatment of PLD/CLD exists which has created divergent treatment approaches by clinicians [3,7,18,19,20]. While several potential disease mechanisms, including persistent infection, immune dysregulation, tissue damage, and the presence of another untreated tick-transmitted infection have been proposed [3,20], the pathophysiology underlying the symptoms and signs of PLD/CLD is still unknown. The development of reliable diagnostic testing that can identify an ongoing infection or demonstrate bacterial eradication remains an unattained goal [3]. Antibiotic retreatment trials of patients with PLD/CLD have been hampered by the lack of a reliable diagnostic markers to determine both entry criteria and treatment outcomes. All of the four NIH-sponsored clinical retreatment trials were quite small [21,22,23]; yet, two found a beneficial treatment effect on severe fatigue [22,23]. 

Patients with PLD/CLD often encounter many barriers to care, including insurance coverage, healthcare costs, travel time and distance to obtain care, and availability of care. These access to care issues were the subject of previous patient surveys conducted by LymeDisease.org., resulting in white papers and peer-reviewed publications [5,24,25]. LymeDisease.org is a non-profit organization that addresses the needs of patients with Lyme disease. For more than 10 years, it has conducted and published peer-reviewed studies based on patient reported data from individual surveys as well as from its patient registry, MyLymeData, which has enrolled over 17,000 patients.

Patients with PLD/CLD report diagnostic delays, having to see many doctors before being diagnosed, traveling significant distances to receive care, and incurring greater out-of-pocket healthcare costs [5,24,25]. More than three-quarters (78%) of these patients were not diagnosed within the first 6 months of illness [25]. Most patients see more than four physicians before they are diagnosed, creating delays that may profoundly impact their quality of life. To obtain care, 49% travel more than 50 miles; 31% report traveling 100 miles or more for care [5]. The cost, inconvenience, and work-related impact of traveling these distances may result in many patients foregoing care all together [24]. 

While health care policy decisions frequently emphasize the importance of the patient story, the perspective of clinicians who treat patients with PLD/CLD is often overlooked. The goal of this study was to identify challenges faced by clinicians who provide care for patients with PLD/CLD as well as information regarding their professional qualifications and clinical experience in treating PLD/CLD. LymeDisease.org surveyed clinicians who treat PLD/CLD patients to learn about the challenges they face in providing care to these patients. To our knowledge, this is the first survey conducted on this topic of clinicians who treat PLD/CLD. This paper reports the results of that survey. 

## 2. Materials and Methods

Between 23 September and 1 December 2021, LymeDisease.org conducted a survey of U.S. clinicians who treat PLD/CLD patients. The survey consisted of 30 closed response items that were multiple choice and one open text item that asked “If you have any comments about this survey that you would like to share, please provide them below”. Survey items were developed by LymeDisease.org in consultation with a small group of community clinicians who treat PLD/CLD and have familiarity with the challenges and obstacles clinicians face in providing care. 

The structured survey questions focused on professional characteristics of clinicians who treat PLD/CLD, access to care issues reported by patients in previous surveys, and concerns clinicians have raised in testimony before government hearings and professional conferences over time. These include: challenges faced by those seeking to treat PLD/CLD, supply demand constraints, difficulty providing care under an insurance model of practice, and regulatory and legal challenges. Examples of survey items, include “how many patients with Lyme disease have you treated in your career?”, “what do you think are the major challenges that face clinicians who treat Lyme disease”, “have you ever felt stigmatized or disrespected by professional colleagues because you treat Lyme disease”, and “what processes does your practice have in place regarding billing and insurers for Lyme disease treatment?”. 

Clinician data was submitted anonymously to protect clinician privacy and encourage greater candor in questions related to stigma, medical board inquiries, and other sensitive information.

Respondents who participated in this study were US residents who reported being clinicians who treated Lyme disease. The survey was distributed by LymeDisease.org through a variety of methods including emails to clinicians enrolled in its clinician referral program as well as a broader email outreach to clinicians. One hundred and fifty-five clinicians from 30 states responded to the survey and 45 provided comments in the open text survey item. 

Data was collected using the widely-used survey platform, Survey Monkey, which gathers survey data, tabulates responses, and provides descriptive statistics relevant to the questions asked (for example the total number of respondents and percentage of respondents who selected each item choice).

## 3. Results

Questions and responses fell into three general categories: the qualifications and clinical experience of the clinicians providing care; the nature of their practices; and the challenges to providing care. 

### 3.1. Clinical Qualifications and Experience of Those Who Treat PLD/CLD

Most clinicians treating PLD/CLD patients are medical doctors (MD) or doctors of osteopathy (DO), belong to a variety of medical societies, and have received continuing medical education (CME) training on tick-borne diseases. More specifically:

- 55% are MDs/DOs, 15% are naturopaths with prescription privileges, 12% are nurse practitioners, 6% are physician assistants
- 89% are members of the International Lyme and Associated Diseases Society, 38% belong to a state medical society, and 26% are members of the American Medical Association, American Academy of Family Physicians or the American College of Physicians
- 98% have taken CME courses on tick-borne diseases

**Figure 1:**
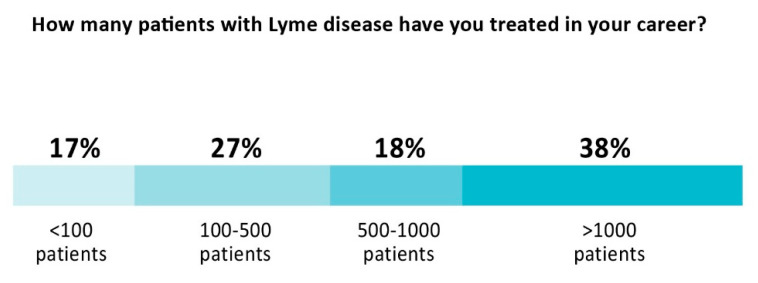
Clinician experience treating patients with PLD/CLD.

Clinicians providing care have extensive experience treating PLD/CLD patients. As Figure 1 below illustrates, more than half (56%) have treated over 500 patients in their career and 38% have treated more than 1000 patients. The majority (57%) dedicated more than half of their practice to Lyme disease, with 19% reporting that they treat Lyme disease exclusively. Almost all (91%) treat using a combination of antibiotics and alternative medicine approaches. Most (85%) do not have hospital privileges.

### 3.2. Nature of PLD/CLD Practices

**Figure 2:**
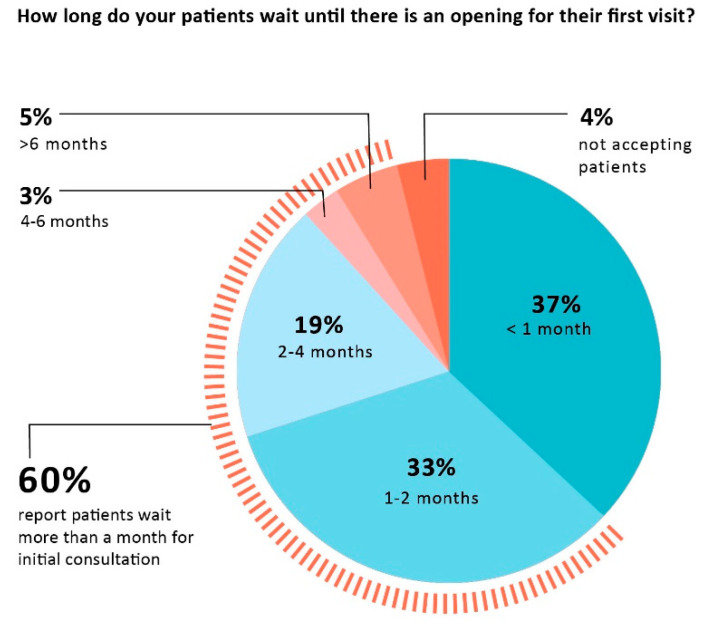
Wait times for initial consultation.

Clinicians report that delays in initial consultations and that caring for patients who come from out-of-state are both common occurrences. Most patients (63%) are not able to book an appointment with their practice within one month for an initial consultation, 33% wait 1–2 months, 19% wait 2–4 months, 8% wait longer than 4 months. Four percent of clinicians are not accepting new patients. Figure 2 shows delays clinicians report for an initial consultation visit. More than a third (38%) report that 25% or more of their practice consists of patients from out of their state of practice. 

Diagnostic and treatment delays are associated with the development of PLD/CLD [6,12], and the subsequent need to consult a clinician who treats PLD/CLD. When asked to identify the three main reasons for delays in patient diagnosis, clinicians specified:

- Inadequate physician education about tick-borne diseases (86%)
- False negative lab test (61%)
- Prior misdiagnosis (52%)
- Lack of EM rash (37%)
- Patient residence in a state believed to be low incidence (25%)
- First disease manifestations were those associated with late disease (21%)
- Public misinformation (16%)
- Patient delay in seeking care (5%)

### 3.3. Challenges of Providing Care

Clinicians reported that they faced significant challenges in providing care to this population including the complex nature of the illness and economic limitations. Clinicians identified the complexity of care (79%), cognitive impairment of patients (57%), and frequent patient calls between scheduled appointments (49%) as barriers to providing care. Some representative open text comments, include:

*“Lyme disease is the hardest diagnosis I treat as each patient responds so differently to therapy; it is hard to know where to start with each one, what will work, what will make them worse, etc. Some just never seem to get better no matter what I do. It is gratifying to see others improve.”*

*“I think the most difficult problem is the cost of providing this amount of complex care on a cash basis. To really review hundreds of records, spend time with the patient and do a proper workup, takes hours. I’d like to see more support for patients and clinicians who chose to help this set of patients.”*

Clinicians reported that length of visits associated with providing such care, administrative burdens and payment issues pose significant challenges that affected the economic feasibility of providing care under a traditional insurance based model. These included extended duration of initial consult and follow-up visits; 25% reported that initial consultations lasted more than 2 h and 44% reported these visits lasted one to two hours. Forty percent reported that follow-up visits lasted one or more hours. Prior authorization of medications (77%), insurance denials (71%), other insurance-related issues (49%), and the inability of patients to pay for out-of-pocket costs (75%) were also identified as practice burdens.

*“I knew that at some point I would be forced to stop taking insurance and move forward on a cash pay only basis. Looking at the numbers, taking commercial insurance for these patients just doesn’t make any sense. I believe that the main issue that causes many of these patients to be without access to care is the amount they need to spend on their practitioners plus the out of pocket costs for out of network testing, labs, and treatment. For most of these patients that is anywhere from $10 to $20K per year. It is a huge burden.”*

**Figure 3:**
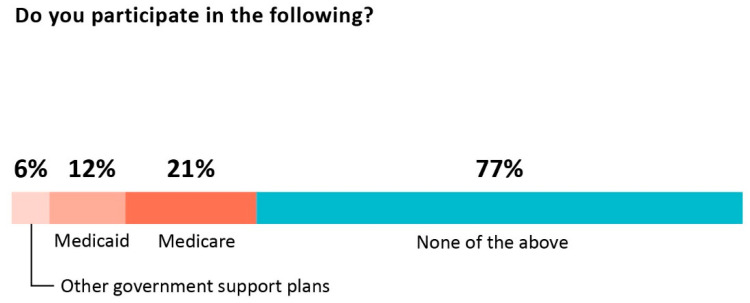
Participation in government supported health plans.

Acceptance of insurance by clinicians was limited, with roughly three quarters of those responding reporting that they did not participate in insurance networks (74%) or directly bill insurers (76%). As described in Figure 3, 77% do not participate in Medicare, Medicaid, or other government supported plans.

Clinicians report that providing care is more difficult because of professional stigma and their exposure to investigations and potential censure by state medical boards, insurers and hospital quality improvement committees. Three-quarters of respondents report having been stigmatized or treated disrespectfully by professional colleagues because they treat Lyme disease. The lack of professional support from colleagues (61%), opposition to treatment of PLD/CLD from some physician organizations (59%), limited ability to share office and practice burdens with other colleagues (20%), and being excluded from participating in insurance networks (11%) were cited as examples of professional marginalization.

Respondents identified regulatory investigations as an important challenge to providing care; 39% report having been reported to a medical board, an insurer, or subjected to a hospital-based quality improvement inquiry. More specifically,

- 19% have been subjected to a medical board inquiry
- 12% have been reported to a medical board by a colleague for treating Lyme disease
- 11% have been reported to a third party payer/insurer
- 11% have been reported to a medical board by a patient
- 10% have been reported to a medical board by an anonymous complainant
- 5% have been reported to a medical board by an insurer
- 4% have been subjected to a hospital-based quality improvement inquiry involving Lyme disease.

*“While my patients are generally very supportive, some of my colleagues have stopped speaking to me and I worry about the medico-legal repercussions of what I do.”*

*“I am anxious about being identified and called out by my colleagues but I feel I must treat patients because so many suffer and cannot find the care they need. I started treating about a year ago, before that I was just diagnosing and referring out. Now Cleveland Clinic refers to me. They diagnose but decline to treat!”*

*“I used to practice in a state where physicians who treat complex patients, including people with chronic Lyme, were specifically targeted by health insurance companies for medical board complaints and other attacks especially when they helped patients other doctors gave up on. Eventually I elected to move to [another state] where there is less interruption of care and more protection of vulnerable patients from predatory insurance entities.”*

Similar concerns were reported as obstacles that impede other clinicians from entering the field. Respondents identified these obstacles: electing to treat a disease that is frequently misunderstood and marginalized professionally (85%), mastery of the subject matter (74%), and diagnostic and therapeutic uncertainty (72%) were major challenges for those seeking to enter the field. Other impediments include the lack of professional support for clinicians treating PLD/CLD (51%) and difficulty identifying peer support (36%).

## 4. Discussion

Access to healthcare, according to the National Academy of Medicine (NAM) can reduce the incidence of preventable diseases, provide early detection and diagnosis of treatable diseases, and reduce chronic disease mortality and morbidity [26]. The first step in obtaining access to healthcare is simply making sure those who need care get into the healthcare system and are able to obtain care [26,27,28]. The NAM defines “access to healthcare” as the timely use of medical care to achieve the best possible outcome: “The most important consideration is whether people have the opportunity for a good outcome—especially in those instances in which medical care can make a difference. When those opportunities are systematically denied to groups in society, there is an access problem that needs to be addressed” [26]. 

Barriers to care can be geographical, financial, or organizational in nature, resulting in a failure to provide needed services [27]. Structural barriers that contribute to poor medical care are driven by factors that fall into three domains: Economic incentives; knowledge, bias, and uncertainty; and power and human relationships [29].

Previous research has found that PLD/CLD patients frequently face significant difficulties in receiving treatment for their illness [5]. This survey suggests that clinicians who treat this population face substantial obstacles when trying to provide PLD/CLD patients with the care they need.

The primary goal of this survey was to identify the challenges faced by clinicians who provide care for patients with PLD/CLD. Clinicians treating patients with PLD/CLD have developed substantial clinical expertise, but encounter multiple clinical, regulatory and financial obstacles to providing care. Fundamentally, the responses paint a picture of structural barriers that are difficult for clinicians to surmount. 

Prior surveys of patients with PLD/CLD suggest that there is a shortage of treating clinicians [5,24,25]. In those surveys, patients reported difficulty finding a knowledgeable clinician and often having to travel significant distances to receive care, with greater out-of-pocket healthcare costs. Clinicians here reported that initial consults in their practices for patients with PLD/CLD were often substantially delayed and more than a third reported that at least 25% of their patients with PLD/CLD came from outside their state of practice. The difficulty in accessing timely local care identified in both the patient and physician surveys point to a supply demand crisis, with patient demand outstripping the available supply of treating clinicians.

Findings from a 2010 survey of Connecticut physicians confirm this disparity between supply and demand [30]. In that survey, primary care specialists – family medicine, general internists and pediatricians, were provided a definition of chronic Lyme disease and then asked “to estimate the number of their primary care patients in whom they had diagnosed and treated Lyme disease, chronic Lyme disease, or both in the last 3 years.” Despite Connecticut being highly endemic for Lyme disease, only 2.1% of the 285 respondents reported that they treated patients with chronic Lyme disease.

Clinician respondents corroborated patient reports of prior misdiagnoses with 52% of PLD/CLD clinicians citing it as a primary reason for delayed treatment. In a previous study using a different sample of patients enrolled in MyLymeData, the majority of patients (72%) reported being misdiagnosed with another condition prior to their Lyme diagnosis [31]. In a retrospective case series of patients presenting with potential early Lyme disease, 54% of Lyme disease patients who presented without a rash are misdiagnosed [32]. Hence, misdiagnosis should be considered a major risk factor in the development of PLD/CLD.

Unlike most health care, PLD/CLD clinicians provide care for a disease that is frequently misunderstood and marginalized. One expert defines stigma and summarizes its impact in health care as: “Stigma is a powerful social process that is characterized by labeling, stereotyping, and separation, leading to status loss and discrimination, all occurring in the context of power” [33].

Three-quarters of respondents report experiencing professional stigmatization, including the threat of censure or worse from medical boards, insurers and hospital quality improvement committees. The heightened professional threat to treating clinicians is illustrated by the fact that other recognized institutions outside the community diagnose, but refuse to treat. Clinician marginalization can have profound effects on those who experience it, including: exclusion from insurance networks; limited opportunities to share office space and resources, on-call and hospital duties; and the stifling of collegial information sharing regarding advances in research as well as clinical observations and breakthroughs. Some of the ramifications of stigma substantially increase the cost of providing care. These effects are reflected in the challenges respondents identified here. One way clinician respondents avoid targeting by insurance companies and others is by not participating in insurance networks or Medicare and Medicaid. 

The risk of being targeted by payers is one impediment to participating in insurance-based clinical practice, another is that reimbursement for PLD/CLD does not reflect the skill or time allocations required to provide care. Because the traditional economic model of medical provider compensation relies heavily on insurance coverage and working in-network, when clinicians opt out of the insurance model, it shifts the economic burden of care to patients. It is not surprising that clinicians identify the ability of patients to pay out-of-pocket as a major challenge to providing care. Patients face structural barriers to care when they are unable to obtain care from their usual provider or must seek care from sources that do not accept insurance due to the complexity of the care required. Their ability to obtain care, as well as the financial impact on their lives, can be significant. Medical costs and income loss play a significant role in personal bankruptcies [34].

## 5. Strengths and Limitations

The primary strengths of this study are that it is the first study to investigate access to care issues in PLD/CLD from the clinician perspective and it does so using real world data. The combination of structured questions and the open comment sections gathered both quantitative and qualitative data, with the latter providing useful framing for the former. There are limitations to this study. Respondents were recruited to participate and are not a random sample of PLD/CLD clinicians. The data is self-reported and observational. 

## 6. Conclusions

Given the dramatic rise in cases of Lyme disease, the demand for PLD/CLD providers will continue to grow. The limited availability of knowledgeable clinicians and the growing population of patients with PLD/CLD has produced a significant supply and demand imbalance that creates an access to care issue for patients that urgently needs to be addressed. 

This survey of treating clinicians identified several structural barriers that contribute to this problem including professional stigma and regulatory and insurance constraints. Clinician-encountered barriers may be powerful disincentives for providing care to patients with PLD/CLD that make it difficult to retain and recruit clinicians. Initial steps to remove barriers to providing care and addressing the supply and demand imbalance in PLD/CLD include:

- Improving clinician education regarding PLD/CLD to emphasize the uncertainty that exists regarding its pathogenesis, diagnosis and treatment along with the existence of divergent approaches to care;
- Setting a more professional tone towards treating clinicians to reduce stigma, marginalization, and regulatory burdens; and
- Promoting innovative insurance reimbursement models that account for the complexity of care and time commitment necessary to provide care will.

Resolving the supply/demand imbalance is essential for ensuring that patients to receive the care they need to become well.

## Data Availability

The Data used in the preparation of this article were obtained from the survey conducting by LymeDisease.org and are available on request from the lead author.
